# Methemoglobinemia in the Era of COVID-19: A Potential Confounder of Glycemic Control

**DOI:** 10.1210/jcemcr/luad035

**Published:** 2023-03-24

**Authors:** Benjamin M Ascherman, Kolton Smith, Sarah L Fishman

**Affiliations:** Department of Medicine, Lenox Hill Hospital, Northwell Health, New York, USA; Department of Medicine, Lenox Hill Hospital, Northwell Health, New York, USA; Division of Endocrinology, Lenox Hill Hospital, Northwell Health, New York, USA; Division of Endocrinology, Lenox Hill Hospital, Northwell Health, New York, USA

**Keywords:** methemoglobinemia, COVID-19, hemoglobin, glucose

## Abstract

Glycated hemoglobin A_1c_ (HbA_1c_) is frequently used as a measure of glycemic control but can be inaccurate in certain clinical scenarios leading to poor estimates of insulin requirements. We present the case of a 76-year-old man with diabetes and COVID infection. HbA_1c_ was measured at 5.7%, though the patient reported home glucose readings of 200 to 300 mg/dL (11.1-16.65 mmol/L). Pulse oximetry on presentation was 50% to 60%, which initially improved to 93% with supplemental oxygen of 15 L via nonrebreather face mask. Treatment with remdesivir and dexamethasone was initiated, but the patient was again found to have low oxygen saturations requiring bilevel positive airway pressure and transfer to the intensive care unit. The patient was started on 1.1 U/kg of insulin daily in a basal-bolus regimen. The patient developed severe hyperglycemia requiring 2.4 U/kg to achieve glycemic control. Co-oximeter analysis of an arterial blood gas sample revealed methemoglobinemia. Exchange transfusion was performed with clinical improvement. Subsequent measurement of fructosamine was 360 umol/L (360 000 µmol/L), correlating with reported home glucose measurements. Methemoglobinemia may impair glycation of hemoglobin or interfere with measurement of HbA_1c_, thereby compromising the use of this molecule as a marker for glycemic control in patients with this condition.

## Introduction

Infection with the novel coronavirus SARS-CoV2 can have numerous effects on various organ systems. In patients with diabetes, interactions between the virus and host can lead to profound inflammatory and immune responses resulting in labile glycemic control [1]. Measurement of glycated hemoglobin A_1c_ (HbA_1c_) is fundamental in estimating glycemic control. An understanding of the strengths and weaknesses of this serum marker, as well as its role alongside other clinical and laboratory findings, is critical in guiding the management of diabetes among COVID-infected patients. We present a case of COVID infection in a patient with diabetes and underlying methemoglobinemia.

## Case Presentation

A 76-year-old man with type 2 diabetes mellitus was admitted after collapsing at home, 2 weeks after testing positive for COVID-19. He was found to have a blood-oxygen saturation level of 50% to 60% on room air by emergency services. Past medical history was notable for chronic obstructive pulmonary disease, asthma, Parkinson disease, and coronary artery disease. Medications included fluticasone propionate/salmeterol, umeclidinium, montelukast, fluticasone nasal spray, albuterol inhaler, hydrochlorothiazide, lisinopril, nifedipine, tamsulosin, atorvastatin, aspirin, carbidopa-levodopa, primidone, gabapentin, and omeprazole. Regarding his diabetes, he reported using 75 units of insulin (1.4 U/kg, 30 long-acting and 15 short-acting pre-meal) daily with home glucose measurements 3 times daily in the 200 to 300 mg/dL (11.1-16.65 mmol/L) range. He reported a 20-year history of diabetes, for which he followed up with an endocrinologist at an outside institution and had been prescribed insulin for “many years.” He denied polyuria, polydipsia, recent weight loss, blurry vision, symptoms of peripheral neuropathy, or known history of hypoglycemic episodes. He was a former smoker and denied excessive alcohol consumption.

## Diagnostic Assessment

**Figure 1.**
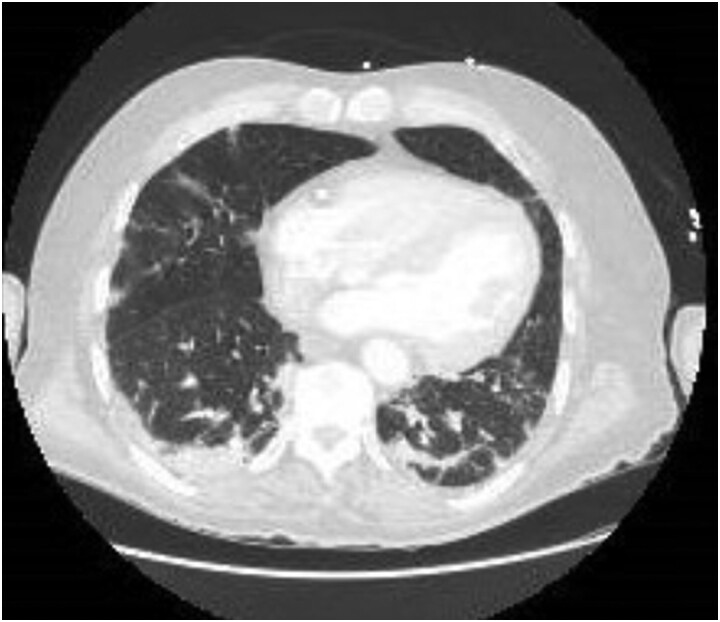
Computed tomography angiogram consistent with COVID pneumonia.

Exam on arrival to the COVID unit was notable for a temperature of 98° F (36.66° C), heart rate of 99 bpm, respiratory rate of 20 breaths/minute, blood-oxygen saturation level of 93% on 15 L non-rebreather mask, weight 54 kg (119.05 lb) and body mass index of 22. He appeared uncomfortable, with dry mucous membranes, and had crackles in the posterior lung fields. Laboratory evaluation revealed a glucose level of 122 mg/dL (6.77 mmol/L), HbA^1c^ of 5.7%, hemoglobin 12.3 g/dL (123 g/L), and venous blood gas with oxygen partial pressure less than 15 mm Hg. Computed tomography angiogram of the chest was consistent with COVID pneumonia without evidence of pulmonary embolism (Fig. 1).

## Treatment

**Figure 2.**
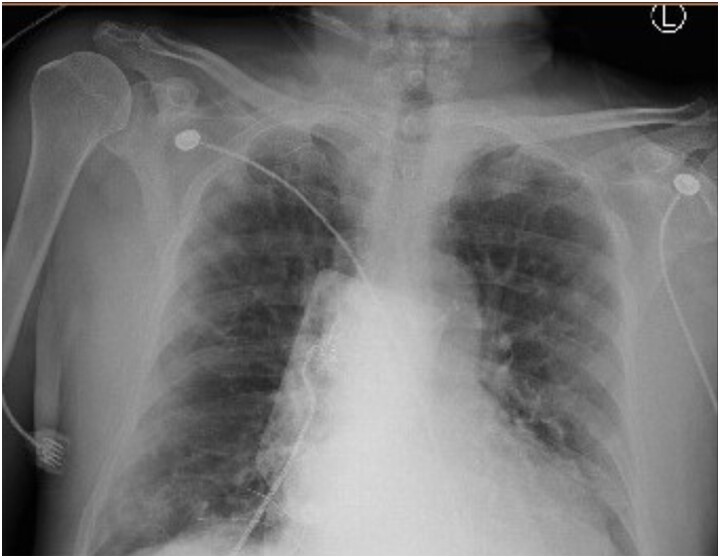
Chest x-ray showing bilateral peripheral patchy infiltrates consistent with known COVID-19 infection.

The elevated blood glucose readings in the setting of an abnormally low HbA_1c_ prompted the internal medicine team to consult endocrinology the morning after admission. In light of the patient’s normal body mass index and poor oral intake in his acutely ill state, he was started on 60 units daily of insulin (1.1 U/kg) administered as a basal-bolus regimen. Overnight the patient's oxygen requirements declined transiently; however, he then developed shortness of breath and demonstrated oxygen saturation levels of 50% to 60%. He was placed on bilevel positive airway pressure and transferred to the intensive care unit. Chest x-ray failed to reveal additional findings to explain his respiratory deterioration (Fig. 2). The arterial blood gas sample was noted to be dark brown, prompting measurement of a co-oximeter profile revealing pH 7.41, partial pressure of carbon dioxide 37 mm Hg, partial pressure of oxygen 177 mm Hg, bicarbonate 23 mmol/L (12.3 mmol/L), total hemoglobin 8.2 g/dL (82 g/L), oxyhemoglobin 76% (94%-100%), carboxyhemoglobin 1.9% (≤ 1.5%), and methemoglobin 21.0% (≤ 1.5%) with an oxygen saturation of 99% (95%-100%). The diagnosis of methemoglobinemia was made around 24 hours after initial presentation to the hospital. The patient underwent exchange transfusion with subsequent normalization of methemoglobinemia, and improvement of oxygenation on room air. Serum fructosamine level was found to be 360 umol/L (360 000 µmol/L), suggesting an average glucose level of 212 mg/dL (11.7 mmol/L) over the previous 14 days. Serum fructosamine level was ordered at the behest of the endocrinology team in light of the new diagnosis of methemoglobinemia and concern for its confounding of the HbA_1c_. Given the swift recognition of the invalidity of this patient’s HbA_1c_, this data point was not used in the consideration of insulin dosing beyond the first day of hospitalization.

## Outcome and Follow-up

**Figure 3.**
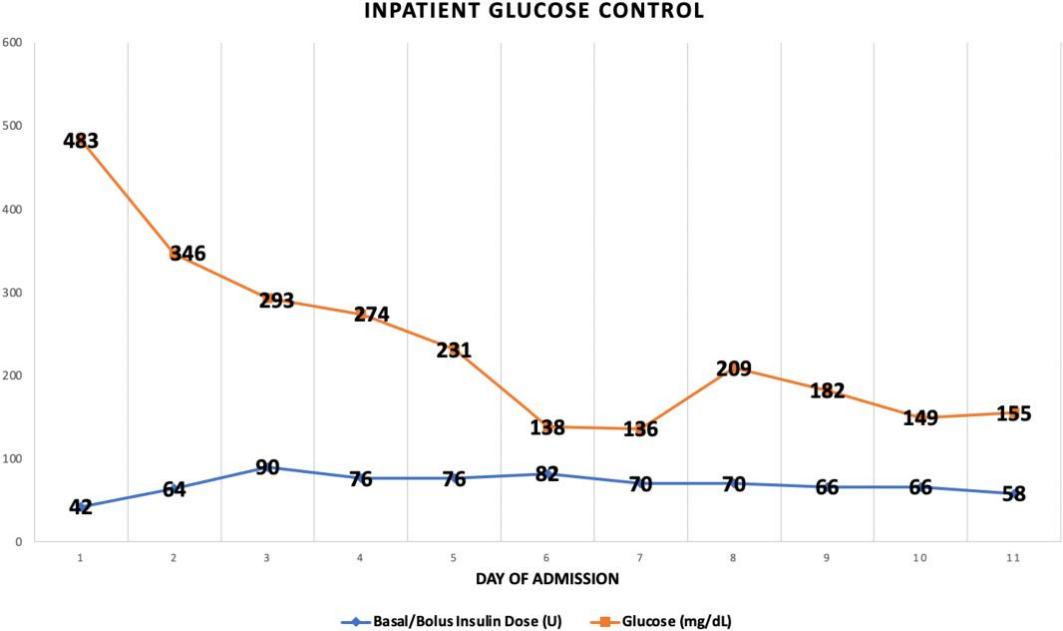
Graph illustrating the blood sugars and correlating basal/bolus insulin dosages each day of hospitalization.

Throughout the patient’s hospitalization, glucose values were consistently elevated and insulin doses were escalated to achieve a target glucose of 100 to 180 mg/dL (5.55-9.99 mmol/L) (Fig. 3). Maximum insulin requirements were 132 units/day (∼2.5 u/kg, basal-bolus plus corrective insulin), which declined after discontinuation of dexamethasone. The patient's respiratory status improved, and he was discharged on his preadmission insulin regimen.

## Discussion

HbA_1c_ has become a standard tool for diabetes screening and prior glycemic control. However, HbA_1c_ can be affected by numerous factors unrelated to glucose metabolism, leading to unreliable prediction of glycemic control. Anemia, hemoglobinopathies, hemolysis, hemorrhage, recent transfusion, certain drugs, uremia, splenomegaly, and other disruptors of hemoglobin turnover and structure can affect HbA1c levels. Other biomarkers such as 1,5-anhydroglucitol (1,5-AG), fructosamine, and glycated albumin (GA) can also be used to estimate glycemic control. Fructosamine measurement in our patient was 360 umol/L (360 000 µmol/L), suggesting an average glucose of 212 mg/dL (11.7 mmol/L) or HbA_1c_ of 9%, well above the estimated mean glucose of 117 mg/dL (6.49 mmol/L) predicted by the measured HbA_1c_ of 5.7%, and likely a better estimate of the patient's true glycemic control [2]. The stark contrast in glycemic control depicted by fructosamine vs HbA_1c_ highlights how different markers can provide different clinical pictures. At least part of this difference can be explained by the fact that glycation of the hemoglobin molecules occurs within red blood cells, whereas glycation of serum proteins such as fructosamine occurs in the extracellular compartment [2]. In methemoglobinemia, oxidation of the iron atoms in hemoglobin from the ferrous (Fe^2+^) to ferric (Fe^3+^) state produces a structural change in hemoglobin that impairs its ability to offload oxygen to tissues and can result in tissue ischemia. The methemoglobin molecule is thought to interfere with the liquid chromatography assay used to measure HbA_1c_, producing falsely low values [3]. Different laboratory techniques used to measure HbA_1c_ vs fructosamine (in this case, turbidimetric inhibition immunoassay, or TINIA, for HbA_1c_, vs colorimetric method for fructosamine) may be affected in different ways by methemoglobinemia, thus explaining the discrepancy in this patient. Methemoglobin levels may be increased during sepsis, as are levels of nitrites and nitrates, both of which are markers of nitric oxide production [4]. When exposed to oxyhemoglobin in red blood cells, nitric oxide oxidizes iron to its ferric state to produce methemoglobin and nitrate, a process that may be exacerbated by increased levels of oxidative stress during COVID infection [5]. Drug-induced methemoglobinemia may also have contributed in this case. Further history revealed that the patient had recently undergone vitamin C infusion, a vitamin with both antioxidant and pro-oxidant effects, which may increase methemoglobin production in humans [6]. Hydroxychloroquine, a drug closely related to chloroquine that is a known trigger for methemoglobinemia, has been used to prevent COVID infection and may also play a role in drug-induced methemoglobin production, which may be enhanced by the oxidative stress of the infection described earlier [7, 8]. Indeed, numerous reports of unexplained methemoglobinemia in COVID patients with persistent hypoxia have been reported, highlighting the need for further research to better understand these phenomena and how methemoglobinemia may affect both true HbA_1c_ levels and measurement of HbA_1c_ in the laboratory [9]. As in other infections, poor glycemic control can increase complication rates and worsen prognoses in COVID-infected patients [10]. Management of COVID in patients with diabetes is especially challenging as treatment often includes the use of systemic steroids, which can markedly exacerbate hyperglycemia. This case highlights the importance of taking a holistic approach when evaluating glycemic control in patients with diabetes and COVID. Our patient's reported elevated home glucose measurements served as an important clue that the measured HbA_1c_ of 5.7% was likely an unreliable predictor of glycemic control. An understanding of both the utilities and pitfalls of markers of glycemic control, especially HbA_1c_, is important in estimating insulin requirements for admitted patients, and can help improve the diagnosis and management of underlying disorders such as methemoglobinemia.

## Learning Points

- HbA_1c_ can be inaccurate in certain clinical scenarios leading to poor estimates of insulin requirements.
- Methemoglobinemia may affect the ability of hemoglobin to accept glycosylation, leading to blood glucose measurements that contradict A_1c_ values.
- Alternate methods of the measurement of blood glucose control, such as serum fructosamine levels, are useful in clinical scenarios in which the A_1c_ may be inaccurate.

## Contributors

All authors made individual contributions to authorship. B.A. and S.F. were involved in the diagnosis and management of this patient and manuscript submission. B.A. is responsible for the histopathology section and preparation of histology images. K.S. is responsible for the chart review of the case as well as editing of the manuscript and final preparation/writing for submission of the manuscript. All authors reviewed and approved the final draft.

## Data Availability

Original data generated and analyzed during this study are included in this published article or in the data repositories listed in “References.”

## Abbreviations

HbA_1c_: glycated hemoglobin A_1c_
